# Short-Term Photovoltaic Power Forecasting Based on an Improved Zebra Optimization Algorithm—Stochastic Configuration Network

**DOI:** 10.3390/s25113378

**Published:** 2025-05-27

**Authors:** Yonggang Wang, Wenpeng Li, Haoran Chen, Yuanchu Ma, Bingbing Yu, Yadong Yu

**Affiliations:** School of Information and Electrical Engineering, Shenyang Agricultural University, Shenyang 110866, China; wygvern@syau.edu.cn (Y.W.); liwenpeng@stu.syau.edu.cn (W.L.); 2023149004@stu.syau.edu.cn (Y.M.); yubingbing@stu.syau.edu.cn (B.Y.); yuyadong@stu.syau.edu.cn (Y.Y.)

**Keywords:** stochastic configuration network, zebra optimization algorithm, photovoltaic power, short-term photovoltaic power forecasting

## Abstract

The output of photovoltaic (PV) power generation systems remains uncertain primarily due to the uncontrollable nature of weather conditions, which may introduce disturbances to the power grid upon integrating PV systems. Accurate short-term PV power forecasting is an essential approach for ensuring the stability of the power system. The paper proposes a short-term PV power forecasting model based on improved zebra optimization algorithm (IZOA)-stochastic configuration network (SCN). First, the historical PV data are divided into three weather patterns, effectively reducing the uncertainty of PV power. Second, a prediction model based on SCN is developed. To enhance the forecasting model’s accuracy even further, the IZOA is introduced to optimize the key parameters of the SCN. Finally, IZOA-SCN is employed for short-term PV power through various weather patterns. Experiment results show that the proposed method significantly improves the prediction accuracy in contrast to other comparison models.

## 1. Introduction

Confronted with the challenges of fossil fuel depletion and the deteriorating environmental conditions, the requirement for renewable energy becomes ever more vital. Among renewable energy resources, photovoltaic (PV) energy, as an effective substitute for electricity produced from fossil fuels, has recently garnered considerable attention due to its abundance, inexhaustibility, and cleanliness [1]. However, PV energy is affected by a multitude of meteorological variables, resulting in fluctuations and nonlinearities. Therefore, integrating large-scale PV energy into the grid may significantly impact the reliability and security of the power grid [2]. Precise PV power forecasting can alleviate its effects on the grid, making it crucial to enhance prediction accuracy.

PV power forecasting is generally divided into long-term, medium-term, short-term, and ultra-short-term forecasting based on different time scales [3]. Long-term PV power forecasting mainly balances the supply and demand relationship between power development, large-scale construction, and financial investment [4]. The forecasting of PV power in the medium term is chiefly employed for the maintenance and management of solar power stations [5]. Short-term PV power forecasting serves as a vital cornerstone for developing generation plans, trading strategies, and scheduling frameworks in the power system [6]. The forecasting of ultra-short-term PV power is predominantly utilized for real-time management of the power grid [7].

The main methods for forecasting PV power fall into three distinct categories: physical models, statistical models, and artificial intelligence (AI) models [8]. Physical models are based on the solar radiation transfer equation to forecast the PV power. However, to improve the accuracy of PV power forecasting, it is necessary to acquire complicated meteorological data and set reasonable parameters for the physical models [9], making their application difficult in practice [10]. Statistical models are based on creating a mapping connection between historical data and actual data to predict PV generation output for the next stage. Statistical models include time series analysis, grey theory, and regression analysis. Literature [11] employed a simple autoregressive integrated moving average (ARIMA) method for PV power forecasting, which demonstrates remarkable forecasting accuracy. Literature [12] applied time series analysis to predict PV power, demonstrating relatively accurate prediction results on smooth datasets. However, due to the nonlinear and fluctuating nature of PV generation, statistical models struggle to accurately capture nonlinear relationships in the face of large amounts of historical data, thereby leading to significant prediction errors.

Compared to statistical models, AI models demonstrate remarkable ability to fit nonlinear relationships and exhibit robust learning abilities. Therefore, scholars have progressively embraced AI models for the forecasting of PV power generation. The literature [13] employed support vector machines (SVM) and weather classification for PV power prediction, marking one of the early integrations of weather data with neural network methods for this purpose. However, the support vectors in the SVM algorithm are determined by quadratic programming, which results in significant time consumption when handling large-scale data. To address these issues, an increasing number of researchers are turning to novel neural network approaches. The literature [14] applied long short-term memory (LSTM) neural networks for short-term PV power prediction in large-scale solar power stations. Simulation results demonstrate that LSTM achieves superior predictive precision in the context of handling large-scale data. However, as the network becomes more deeply trained with an increasing number of parameters, longer training times and elevated overfitting may arise. Consequently, this reduces the effectiveness of the network in capturing long-term data features. In response to these challenges, researchers have embarked on exploring advanced techniques for feature extraction. In the literature [15], convolutional neural networks (CNNs) were introduced for their exceptional capability in capturing intricate data features. Furthermore, the CNN-LSTM hybrid network was established by harnessing CNN’s convolutional layers to extract features prior to their input into LSTM, effectively addressing the inherent challenges of standalone LSTM networks in capturing long-term data features. With the gradual development of combined models and the increasing number of training parameters, each training session incurs significant time and computational costs. To address this issue, researchers have begun to adopt surrogate models [16]. In the literature [17], surrogate-assisted optimization effectively reduced the impact load on the anti-ship missile body configuration. In the literature [18], surrogate-based modeling (SBM) and surrogate-based optimization (SBO) were employed in large-scale satellite swarm reconfiguration, significantly reducing computational costs while achieving superior optimization results. The emergence of this method has also enabled its application in the field of PV power forecasting. The literature [19] introduced a PV prediction model that combines gated recurrent unit (GRU) and K-means cluster, attaining greater prediction precision and less training time relative to LSTM. To capture environmental factors that exhibit close correlation with PV power, the literature [20] proposed a hybrid prediction model based on wavelet packet decomposition (WPD) and LSTM. Compared to LSTM, GRU, and RNN models, the predictive accuracy has been improved across all four seasons. Due to the strong feature extraction capability of AI methods, their application in hard-to-reach and difficult-to-extract deposits has become feasible [21]. In the field of short-term PV power forecasting, prediction models often exhibit poor convergence due to the significant influence of weather fluctuations on PV output and the presence of non-stationary data, making it difficult for the models to capture key features. When combined models are employed, coordination issues among the sub-models can also lead to convergence difficulties. Additionally, combined models typically involve a large number of parameters, resulting in high computational costs for training iteration and increasing the risk of gradient vanishing or explosion problems.

To enhance the rate of convergence and learning performance, stochastic algorithms allow the stochastic assignment of weights and biases in neural networks, demonstrating notable efficacy [22,23]. Consequently, stochastic weighted networks are employed in PV generation prediction. The literature [24] proposed a PV power interval prediction model combining extreme learning machine (ELM) with fuzzy C-Means clustering (FCM) regarding the consequences of diverse weather patterns on prediction accuracy. The random vector functional link (RVFL) network is a randomized variant of a single-hidden-layer feedforward neural (SLFN) network. In the structure of the RVFL network, the input layer and the output layer are directly linked, with weights being randomly assigned within a specified range [25]. The literature [26] integrated a seasonal autoregressive integrated moving average (SARIMA)-RVFL network hybrid model into ultra-short-term PV power prediction incorporating wavelet decomposition to reduce the volatility of PV power. In the literature [27], the mayfly optimization (MO) is utilized to optimize the parameters of the RVFL network, aiming to enhance prediction accuracy for PV power forecasting. Although the RVFL network’s construction process is highly efficient, its universal approximation property may not be ensured if the scope is improperly set or the random parameters are inappropriately assigned [28].

To ensure the universal approximation property of models, the literature [28] proposed a stochastic configuration network (SCN), which is a node-incremental neural network with a supervisory mechanism. It adaptively generates hidden layer nodes and constructs the network structure using random algorithms, possessing advantages such as fast modeling speed and strong approximation ability. The SCN algorithm has gradually gained application within academic research across a wide range of domains in recent years, due to its rapid learning efficiency and data-driven characteristics. The literature [29] proposed an ensemble learning SCN for probabilistic forecasting of PV electricity generation. By combining bagging with SCN, RVFL, and ELM, the proposed method conducted confidence evaluation for each algorithm and averaged the confidence outputs of each algorithm to obtain the final output. The literature [30] proposed a block increment-based SCN (BSCN) and applied it to industrial modeling. This approach constructs a block increment learning framework through batch allocation and the constraint of stochastic parameters. It is important to emphasize that the performance of SCN is highly susceptible to the configuration of parameters. Given that the hyperparameters of SCN are frequently selected manually, it leads to suboptimal performance of the constructed model. Consequently, it is essential to adopt an effective methodology to optimize the hyperparameters of SCN to improve the precision of the model.

Zebra optimization algorithm (ZOA) is a novel bio-inspired algorithm proposed in 2022 [31]. This algorithm draws inspiration from the natural behaviors of zebras, simulating their foraging behavior and predator defense behavior. In contrast to well-known optimization algorithms such as particle swarm optimization (PSO), marine predator algorithm (MPA), and whale optimization algorithm (WOA), ZOA has the advantages of faster convergence speed and fewer parameters to set. The literature [32] used the ZOA algorithm to optimize the weight parameters of residual network (ResNet)-CNN to enhance prediction performance. The literature [33] proposed a ZOA-SNN hybrid model controller to meet load power demands, reduce harmonics, and improve power regulation in PV systems. However, like common bio-inspired algorithms, ZOA is also prone to the issue of easily falling into local optima, leading to the optimized objective parameters being suboptimal solutions, which might adversely influence the performance of the optimization model. In this paper, an improved zebra optimization algorithm (IZOA) is introduced, aiming to enhance the search capabilities of ZOA while avoiding its convergence to local optima. The several novel innovations and significant contributions of this paper are detailed below:

(1) To mitigate the impact of weather-induced fluctuations, K-means clustering is utilized based on global horizontal irradiance (GHI) to classify the data. Additionally, by employing Pearson correlation analysis, meteorological factors with high correlation to PV output are selected as model inputs.
(2) To avoid falling into local optima and to improve search efficiency, an IZOA is proposed in this paper, integrating the opposition-based differential evolution (ODE), Chebyshev chaotic mapping, and nonlinear decreasing strategy into ZOA to improve the convergence performance.
(3) This paper applies SCN to the domain of PV power forecasting and utilizes IZOA to optimize the critical parameters of SCN. Simulations exhibit that generalization capabilities of the SCN model are enhanced, thus enhancing predictive accuracy.

## 2. Related Work

### 2.1. Deep Neural Network

Deep Neural Network (DNN) is an artificial neural network characterized by its multi-layered architecture. As the number of neural network layers increases, the DNN may encounter the vanishing gradient during backpropagation due to cumulative attenuation. Moreover, increasing the depth of the network leads to a proliferation of parameters, which can adversely affect computational efficiency. In DNNs, initial parameters are typically obtained through data-independent random initialization methods, where the uncertainty in these parameter values may impact network convergence. To address these challenges, this paper employs stochastic configuration network (SCN) as a short-term photovoltaic forecasting model.

### 2.2. Stochastic Configuration Network

**Figure 1:**
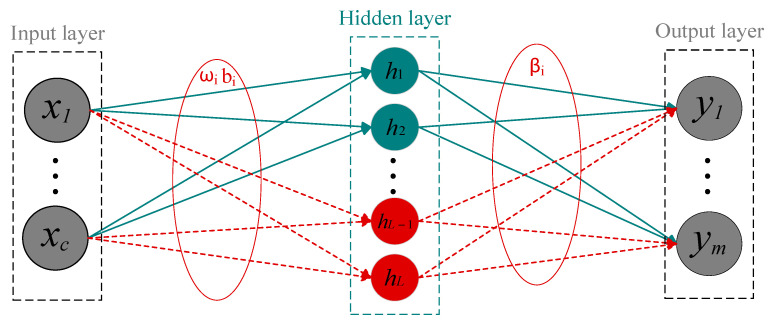
The SCN model structure.

Unlike DNN, SCN starts with a small network containing a single hidden layer with few nodes and gradually increases the number of hidden layer nodes, randomly configuring the input weights and biases of the hidden layer nodes within adjustable ranges. The configuration of parameters needs to be related to the given training samples, rather than completely relying on the distribution or range parameter settings of random weights and biases. The output weights are calculated using the least squares method until the network reaches an acceptable error. The structure is illustrated in Figure 1.

Assuming *L* − 1 hidden layer nodes are generated in a SCN model. Given input $X=\left[x_{1},x_{2},\cdots {,x}_{i},\cdots ,x_{c}\right]$, where $x_{i}={\left[x_{i,1},x_{i,2},\ldots ,x_{i,n}\right]}^{T}$, *c* represents the count of input features, *n* denotes the count of training samples. The corresponding output $Y=\left[y_{1},y_{2},\cdots ,y_{m}\right]$, where $y_{i}={\left[y_{i,1},y_{i,2},\ldots ,y_{i,m}\right]}^{T}$.

The output of the *L*-th hidden node is represented by Equation (1).

(1) $$h_{L}=g_{L}\left(w_{L}^{T}X+b_{L}\right)$$

(2) $$w_{L}=\lambda \times \left(2\times rand\left(n,T_{max}\right)-1\right)$$

(3) $$b_{L}=\lambda \times \left(2\times rand\left(1,T_{max}\right)-1\right)$$

In Equation (1), $g_{L}$ is the activation function of the hidden layer, which is set to the sigmoid function in this paper. $w_{L}$ and $b_{L}$ represent the input weights and biases between the input node and the *L*-th hidden layer node, which are randomly assigned within the range $\left[-\lambda ,\;\lambda \right]$. In Equations (2) and (3), λ is a scale factor. $T_{max}$ represents the maximum number of candidate nodes.

The output of the current network is represented by Equation (4).

(4) $$Y_{L}=\sum_{i=1}^{L}\beta_{i}h_{i}$$

In Equation (4), $\beta_{i}=\left[\beta_{i,1},\beta_{i,2},\cdots ,\beta_{i,m}\right]$ denotes the weight representing the output weights between the hidden layers and the output layer.

The corresponding residual error vector before adding the *L*-th new hidden node is formulated as Equation (5).

(5) $$e_{L-1}=Y-Y_{L-1}={\left[e_{L-1,1},e_{L-1,2},\cdots ,e_{L-1,m}\right]}^{T}$$

In Equation (5), Y and $Y_{L-1}$ represents the output of the current network and output of the network before adding the *L*-th new hidden node. $e_{L-1}$ is the corresponding residual error.

The $g_{L}$ is produced in accordance with Equation (6).

(6) $${\langle e_{L-1,q},g_{L}\rangle }^{2}\geq b_{g}^{2}\delta_{L,m}$$

In Equation (6), $\delta_{L,m}=\left(1-r-\mu_{L}\right){\left\|e_{L-1,m}\right\|}^{2}$. $\mu_{L}$ represents a non-negative real sequence, where $\mu_{L}=(1-r)/(1+L)$, and it satisfies $\underset{L\to +\infty }{\lim}\mu_{L}=0$ and $\mu_{L}\leq (1-r)$. Moreover, *r* is a given random parameter in the interval [0, 1]. This study supposed that span (*Γ*) is dense in *L*_2_ space and ∀gϵΓ, $0<\left\|g\right\|<b_{g}$, for some $b_{g}\epsilon \;R^{+}$.

The output weight $\beta_{L,m}$ between the *m*-th output node and the *L*-th hidden layer node is computed by Equation (7)

(7) $$\beta_{L,m}=\frac{\langle e_{L-1,m},g_{L}\rangle }{{\left\|g_{L}\right\|}^{2}}=\frac{e_{L-1,m}^{T}\cdot h_{L}}{h_{L}\cdot h_{L}^{T}}$$

In Equation (7), $e_{L-1,m}$ represents residual error between the *m*-th output node and the *L*-1th hidden layer node, $h_{L}$ denotes output of the *L*-th hidden node, $g_{L}$ is a basic function.

An evaluation indicator is introduced to assess whether the network outputs meet error requirements. The evaluation indicator $\xi_{L,m}$ is defined as shown in Equation (8).

(8) $$\xi_{L,m}=\left(\frac{{{(e}_{L-1,m}^{T}\cdot h_{L})}^{2}}{h_{L}\cdot h_{L}^{T}}-\left(1-r-\mu_{L}\right)e_{L-1,m}^{T}\cdot e_{L-1,m}\right)\geq 0$$

In Equation (8), $\mu_{L}$ represents a non-negative real sequence, where $\mu_{L}=(1-r)/(1+L)$, and it satisfies $\underset{L\to +\infty }{\lim}\mu_{L}=0$ and $\mu_{L}\leq (1-r)$. Moreover, *r* is a given random parameter in the interval [0, 1]. This study supposed that span (*Γ*) is dense in *L*_2_ space and ∀gϵΓ, $0<\left\|g\right\|<b_{g}$, for some $b_{g}\epsilon \;R^{+}$. $e_{L-1}$ is the corresponding residual error.

**Figure 2:**
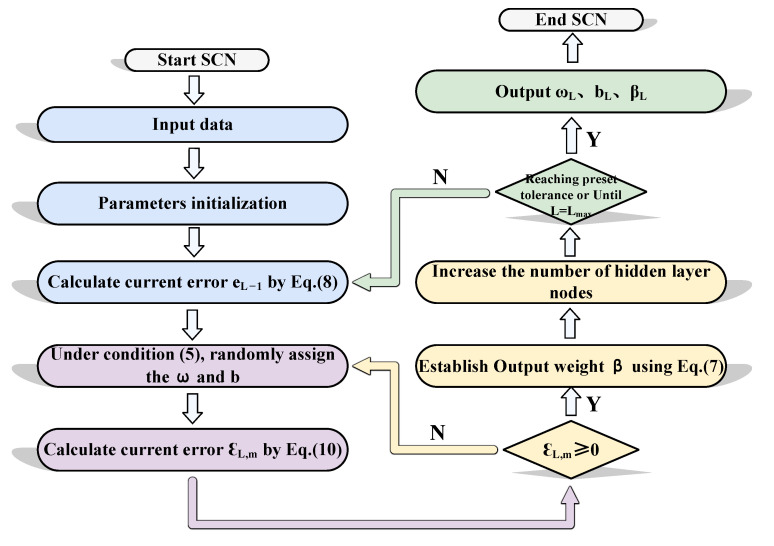
The flow chart of SCN.

As shown in Figure 2, the flow chart of SCN is presented.

### 2.3. Zebra Optimization Algorithm

To solve nonlinear optimization problems, the ZOA primarily encompasses initialization, foraging behavior, and defensive strategies. The specific algorithm of conventional ZOA is as follows.

- Phase 1: Initialization

Each zebra is directly mapped to a decision variable within the search domain. Therefore, each zebra can be viewed as a candidate solution to the question. The number of zebras could be represented by a matrix, as shown in Equation (9).

(9) $$X={\left[\begin{array}{c} X_{1}\\ X_{2}\\ \vdots \\ X_{N} \end{array}\right]}_{N\times m}={\left[\begin{array}{ccccc} x_{1,1} & \cdots & x_{1,j} & \cdots & x_{1,m}\\ \vdots & \ddots & \vdots & ⋰ & \vdots \\ x_{i,1} & \cdots & x_{i,j} & \cdots & x_{i,m}\\ \vdots & ⋰ & \vdots & \ddots & \vdots \\ x_{N,1} & \cdots & x_{n,j} & \cdots & X_{N,m} \end{array}\right]}_{N\times m}$$

In Equation (9), *X* denotes the population of zebras, $X_{i}$ represents the *i*-th zebra, $x_{i,j}$ denotes the value of the *i*-th zebra in the *j*-th dimension.

The original positions of the zebras are randomly assigned within the search domain. ZOA randomly assigns the original positions of zebras using Equation (10).

(10) $$x_{i,j}={la}_{j}+rand\cdot \left(ma_{j}-la_{j}\right)$$

In Equation (10), $la_{j}$ and $ma_{j}$ show the lower and upper bounds within the search domain and *rand* denotes a number chosen at random within the interval [0, 1].

The values of the decision variables are assessed to determine the value of the objective function. Therefore, a fitness function is utilized to evaluate the optimal candidate solution for the corresponding problem, as illustrated in Equation (11).

(11) $$F={\left[\begin{array}{c} F_{1}\\ \vdots \\ F_{i}\\ \vdots \\ F_{N} \end{array}\right]}_{N\times 1}={\left[\begin{array}{c} F\left(X_{1}\right)\\ \vdots \\ F\left(X_{i}\right)\\ \vdots \\ F\left(X_{n}\right) \end{array}\right]}_{N\times 1}$$

In Equation (11), F represents a vector of fitness values, where $F_{i}$ denotes the fitness obtained by the *i*-th zebra.

- Phase 2: Foraging behavior

At this stage, the process of the zebra searching for food is to update the location of the zebra in the search domain. In ZOA, the best individuals in the population are considered as pioneer zebras, guiding others to search for food in different locations. The foraging behavior is described by Equation (12).

(12) $$x_{i,j}^{new,P2}=x_{i,j}+r\cdot \left(PZ_{j}-I\cdot x_{i,j}\right)$$

Here, $x_{i,j}^{new,P2}$ denotes the new value of the *i*-th zebra in the *j*-th dimension, $PZ_{j}$ represents the pioneer zebra within the population. The variable *r* embodies a random value within the interval [0, 1]. $I\in \left\{1,\;2\right\}$, and I=round(1+a), where “*a*” represents a random value spanning [0, 1]. As I increases, a greater number of zebras are moving during the iteration.

After the foraging behavior, zebra updates the position in the search space using Equation (13) as follows:

(13) $$X_{i}=\left\{\begin{array}{c} X_{i}^{new,P2}\;,\;F_{i}^{new,P2}<F_{i}\\ X_{i}\;\;\;\;\;\;\;\;\;,\;else \end{array}\right.$$

where $X_{i}^{new,P2}$ represents the new status of the *i*-th zebra after phase 2. $F_{i}^{new,P2}$ represents the fitness value of the zebra at the new state.

- Phase 3: Defense strategies

At this stage, using the process of zebra defensive behaviors against predators, the position of zebras in the search domain is updated. When faced with large predators, zebras choose the escape strategy; whereas, in the face of smaller predators, they choose the offensive strategy. The likelihood of larger and smaller predators attacking zebras is equal.

The escape strategy is represented using the S1 pattern described in Equation (14), and the offensive strategy is developed according to the S2 pattern described in Equation (14).

(14) $$x_{i,j}^{new,P3}=\left\{\begin{array}{c} S_{1}:x_{i,j}+R\cdot \left(2r-1\right)\cdot \left(1-\frac{t}{T}\right)\cdot x_{i,j},\;\;\;\;\;\;\;\;\;\;\;\;\;\;P_{s}\leq 0.5;\\ S_{2}:x_{i,j}+r\cdot \left({AZ}_{j}-I\cdot x_{i,j}\right),\;\;\;\;\;\;\;\;\;\;\;\;\;\;\;\;\;\;\;\;\;\;\;\;\;\;\;\;else, \end{array}\right.$$

In Equation (14), $x_{i,j}^{new,P3}$ is the updated value for *i*-th zebra within the *j*-th dimension, *T* denotes the maximum number of iterations, *t* represents the current iteration number, and *R* is a constant with a value of 0.01. $P_{s}$ is the probability that a zebra switches between escape or defense strategies, which is a random number in the range [0, 1]. *AZ* represents the state of the zebra being attacked, ${AZ}_{j}$ represents the dimension where it is located.

Position updates are performed using Equation (15).

(15) $$X_{i}=\left\{\begin{array}{c} X_{i}^{new,P3}\;,F_{i}^{new,P3}<F_{i};\\ X_{i}\;,else, \end{array}\right.$$

Here, $X_{i}^{new,P2}$ represents the new status of the *i*-th zebra after phase 3, and $F_{i}^{new,P3}$ denotes the new fitness value of *i*-th zebra at new state.

**Figure 3:**
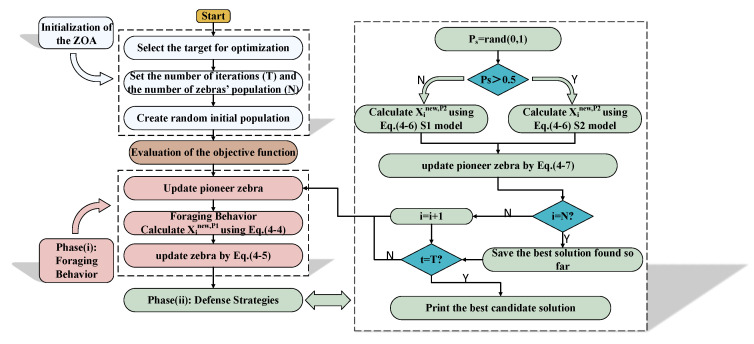
The flow chart of ZOA.

In summary, the flow diagram of ZOA is depicted in Figure 3.

## 3. Improved Zebra Optimization Algorithm (IZOA)

In the previous section, the basic ZOA process is presented. This section will present the improved methods for ZOA. The solution of ZOA is significantly influenced by the initial population distribution, and an uneven initial population distribution can impair its global search capability. Therefore, this paper employs Chebyshev chaotic mapping to improve the initialization phase of ZOA, which can shorten the convergence path, reduce ineffective searches in the early stages, enhance convergence efficiency, and mitigate the risk of local optima. As the search process progresses into the mid-phase, the fitness of the zebra population tends to stabilize or its diversity declines, at which point ZOA still remains susceptible to local optima. To avoid getting trapped in local optima, an opposition-based differential evolution (ODE) is introduced during the mid-phase search. Its mutation and opposition-based population generation mechanisms effectively disturb the current population, enabling ZOA to escape stagnation zones and further reducing the risk of local optima. In the late search phase, to avoid convergence oscillations and premature convergence while preventing ZOA from settling into suboptimal solutions, this paper adopts a nonlinear decreasing strategy to balance local exploitation and global exploration, thereby effectively enhancing convergence precision. In summary, the IZOA proposed in this study integrates Chebyshev chaotic mapping, ODE, and nonlinear decreasing strategy.

### 3.1. Chebyshev Chaotic Mapping

The accuracy of the ZOA solution is affected by the uniformity of the population distribution during initialization [34]. The random initialization positions of the ZOA are predominantly governed by “*rand*” values in Equation (10), which may result in an uneven distribution of the zebra population within the search space. The paper introduces Chebyshev chaotic mapping to substitute the random numbers used in the random initialization of the ZOA, thereby achieving an initial population with high diversity in the solution space. The discrete mapping equation for the Chebyshev chaotic map is given by Equation (16).

(16) $$x_{n-1}=\cos\left(i\cdot arccosx_{n}\right),\;x_{n}\in \left(-1,\;1\right)$$

Here, i denotes the order of the mapping, n represents the number of iterations, and x indicates an element within the sequence of the mapping.

### 3.2. Population Mutation Mechanism Based on Opposition-Based Differential Evolution (ODE)

The paper introduces the ODE [35] to optimize the population of the ZOA. By introducing an opposite population and differential evolution throughout the search procedure, the quality of the ZOA population is enhanced, which in turn improves the search capability.

(a) Opposition-Based Learning (OBL)

In most cases, the optimization algorithm begins with one or several random points. Ideally, the aim of the optimization algorithm is to find the optimal value of the objective function as soon as feasible. The best scenario occurs when the randomly initialized population is close to the optimal value, resulting in rapid convergence. The worst scenario involves the optimal value lying in the opposite search direction of the population, requiring a large number of iterations and a significant amount of time to reach the optimal value. In the absence of any prior information, it is nearly impossible to determine the best initial guess. Therefore, optimization should be conducted in all directions during the search.

The opposite candidate solution $\overset{ˇ}{x}$ is defined according to Equation (17).

(17) $$\overset{ˇ}{x}=a+b-x$$

Here *x* is a real number and $x\in \left[a,b\right].$

Similarly, in solving high-dimensional problems, the opposite candidate solutions can be generalized to *N*-dimensional space, for which the solution is given by Equation (18).

(18) $${\overset{ˇ}{x}}_{k}=a_{k}+b_{k}-x_{k}$$

Here $x_{k}\in \left[a_{k},b_{k}\right]$, $\forall k\in \left\{1,2,\cdots ,N\right\}$, and ${\overset{ˇ}{x}}_{k}$ is the opposite candidate solution.

Let $h\left(x\right)$ be the fitness function, $X=\left(x_{1},x_{2},x_{3},\cdots ,x_{N}\right)$ be the forward candidate solution within the *N*-dimensional space, where $x_{1},x_{2},x_{3},\cdots ,x_{N}$ are real numbers, and $\overset{ˇ}{X}=\left(\overset{ˇ}{x_{1}},\overset{ˇ}{x_{2}},\overset{ˇ}{x_{3}},\cdots ,\overset{ˇ}{x_{N}}\right)$ be the opposite candidate solution. If $h\left(X\right)\leq h\left(\overset{ˇ}{X}\right)$, then replace X with the opposite solution $\overset{ˇ}{X}$, otherwise, retain the X. Therefore, both forward and reverse solutions in ZOA are evaluated simultaneously to keep better solutions for the next iteration.

(b) Differential Evolution (DE)

The differential evolution (DE) primarily consists of mutation, crossover, and selection. Compared to genetic algorithms, DE tends to be more adept at preserving excellent individuals. The specific process of DE is as follows.

(1) Mutation

DE randomly selects two distinct individual vectors from the population, adding its vector difference weighted to another individual to generate a mutation individual $V_{i,G}$, which is calculated by Equation (19).

(19) $$V_{i,G}=X_{r1,G}+F\cdot \left(X_{r2,G}-X_{r3,G}\right)$$

Here, $X_{ri,G}$ is a solution vector in the *G*-th iteration and $i=\left\{1,2,3,\cdots ,N_{p}\right\}$,$\;N_{p}$ represents the population size, $r_{1}$, $r_{2}$, $r_{3}$ are random different integers from i. The parameter $F\in \left[0,2\right]$ is a real constant to adjust the scaling of the differential vector $\left(X_{r2,G}-X_{r3,G}\right)$. A higher value of *F* increases the diversity of the generated population, while a lower value of *F* accelerates the convergence rate.

(2) Crossover

Some genes of the mutated individual are exchanged with the target individual based on a certain probability, thereby producing experimental vector $U_{i,G}$. The binomial cross is described by Equation (20).

(20) $$U_{i,G}=\left\{\begin{array}{c} V_{i,G},\;\;\;\;\;\;\;\;if\;{rand}_{j}\left(0,1\right)\leq CR\;or\;j=P\\ X_{i,G},\;\;\;\;\;\;\;otherwise \end{array}\right.$$

Here, $CR\in \left(0,1\right)$ is the crossover rate, ${rand}_{j}\left(0,1\right)$ represents a random number in the interval (0, 1) generated for the *j*-th dimension, j=1,2,3,⋯,D (where *D* is the dimensionality of the problem). $P\in \left\{1,2,3,\cdots D\right\}$ is a predetermined random integer, employed to ensure that at least one gene is present in the crossover operation from the experimental vector $U_{i,g}$ to the mutation individual $V_{i,G}$.

(3) Selection

After the crossover operation, the greedy strategy is employed to determine which individuals to retain. The fitness values of $U_{i,G}$ and $X_{i,G}$ are compared, and the selection operation is represented by Equation (21).

(21) $$X_{i,G}=\left\{\begin{array}{c} U_{i,G},\;\;\;\;\;\;\;\;\;\;\;\;\;\;\;\;\;\;\;\;\;\;if\;f\left(U_{i,G}\right)\geq f\left(X_{i,G}\right);\\ X_{i,G},\;\;\;\;\;\;\;\;\;\;\;\;\;\;\;\;\;\;\;\;\;\;\;\;\;otherwise \end{array}\right.$$

Here $f\left(*\right)$ represents the fitness function.

**Figure 4:**
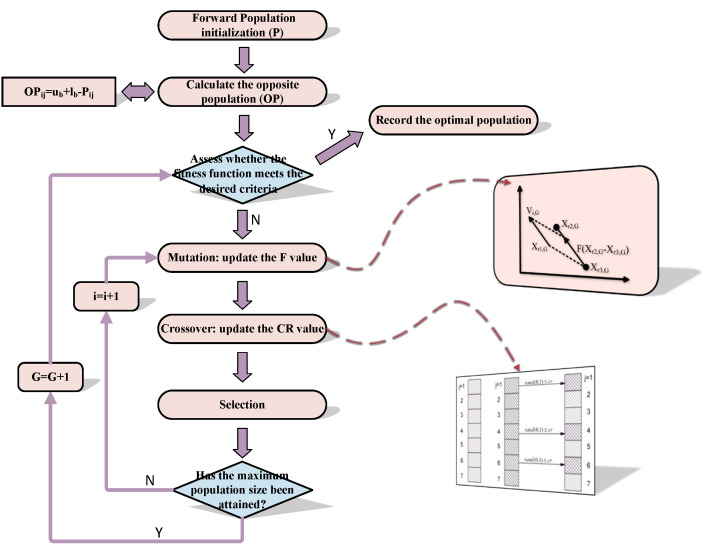
The flow chart of ODE.

In order to improve the performance of the DE and overcome issues of premature convergence, integrating OBL into DE results in the ODE strategy, which can yield more suitable initial candidate solutions. The process of ODE is as follows (see Figure 4):

### 3.3. Nonlinear Decreasing Strategy

In the escape strategy for the defense phase, zebras update their positions utilizing the S1 model as specified in Equation (14). It should be noted that the choice of the *R* value has a profound impact on the position update of the zebras, thereby affecting the optimization accuracy of the ZOA. To improve the optimization capability, the paper introduces a nonlinear decreasing strategy to replace the *R* value in the S1 model, as shown in Equation (22).

(22) $$R_{u}=0.8\cdot e^{-0.01t}$$

The improved formula is presented as Equation (23).

(23) $$x_{i,j}^{new,P2}=\left\{\begin{array}{c} S_{1}:x_{i,j}+R_{u}\cdot \left(2r-1\right)\cdot \left(1-\frac{t}{T}\right)\cdot x_{i,j},\;\;\;\;\;\;\;\;\;\;\;\;\;P_{s}\leq 0.5;\\ S_{2}:x_{i,j}+r\cdot \left({AZ}_{j}-I\cdot x_{i,j}\right),\;\;\;\;\;\;\;\;\;\;\;\;\;\;\;\;\;\;\;\;\;\;\;\;\;\;\;\;else, \end{array}\right.$$

The introduction of the nonlinear decreasing strategy balances the association between global search ability and local search ability, enabling the algorithm to have strong global search capability in the early stages and sophisticated local search capability in the later stages, thereby enhancing the optimization accuracy of the algorithm.

**Figure 5:**
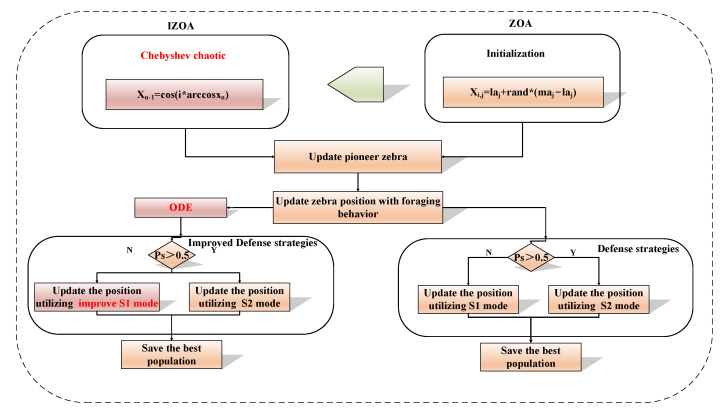
The improved algorithm comparison chart.

In summary, this paper introduces an improvement strategy during the initial, middle, and final stages of the ZOA search. The comparison between the improved and original algorithm flowchart is illustrated in the Figure 5.

IZOA can optimize the main hyperparameter of SCN, including regularization coefficient *r* and scaling factors *λ*. In summary, this article proposes a new PV power generation prediction model IZOA-SCN.

## 4. Establishment of IZOA-SCN Model

The essence of the SCN model revolves around its groundbreaking supervision mechanism, affected by the *r* and *λ*. Specifically, in traditional SCN, the value of *r* affects the contractility of the supervisory mechanism and the compactness of the network, cycling through the non-negative increasing sequence of candidates [0.9, 0.99, 0.999, 0.9999, 0.99999] to ensure the constant efficacy of the supervisory mechanism. The rate of decline of the residual sequence is also influenced by the regularization coefficient *r*. A higher *r* value can lead to a reduction in the learning speed of the network. Conversely, a lower parameter *r* guarantees a swift reduction in the network residuals.

To ensure the requirement of the inequality constraint $\xi_{L,m}\geq 0$, the scaling factors *λ* is selected from the set [0.5, 1, 5, 10, 30, 50, 100, 150, 200, 250], determining the efficiency of finding network parameters and the capacity for generalization. In traditional SCN, *λ* is essential in defining the scope of parameters $w_{L}$ and $b_{L}$. As the scope of *λ* widens, the search domain for weights and thresholds also increased, enhancing the efficiency of finding network parameters and guaranteeing a solution exists for inequality constraint. However, this also increases model complexity, causing the model to be more responsive to questionable data and more prone to overfitting.

The selection of *r* and *λ* should depend on data rather than being fixed to a specific set of values. However, the theoretical foundations for the selection of appropriate hyperparameters remain insufficient. Therefore, to improve the accuracy of short-term PV power prediction of SCN under various weather scenarios, the paper introduces the IZOA to optimize the *λ* and *r*.

The modeling process for IZOA-SCN to PV power forecasting is as follows:

Step 1: Preprocess historical PV power generation data and meteorological data and use Pearson correlation coefficient to select model input features.

Step 2: Utilizing the K-means to cluster PV data, three weather scenarios are obtained.

Step 3: Initialize the parameters of IZOA and SCN: population size (P), maximum number of iterations (T), optimizing the lower bound ($l_{b}$), optimizing the upper bound ($u_{b}$), the maximum number of the hidden nodes ($L_{max}$), tolerance error (Ɛ), maximum number of candidate nodes ($T_{max}$), scaling factors (λ), regularization coefficient (r), initial scaling ratio (F), and crossover rate (CR).

Step 4: Constructing the IZOA-SCN model. The IZOA utilizes *r* and *λ* as population individuals and employs the network residual of SCN as the fitness function to explore the optimal candidate pool sequence of two hyperparameters.

Step 5: Determine the termination condition and finalize the sequence of parameters. Iterations terminate when the tolerance error is met or the maximum number of iterations is reached, obtaining the optimal pool of parameters for *r* and *λ*.

Step 6: Perform short-term PV forecasting and produce the ultimate PV forecasting outcomes.

Step 7: Analyze and focus on the prediction results and related errors.

**Figure 6:**
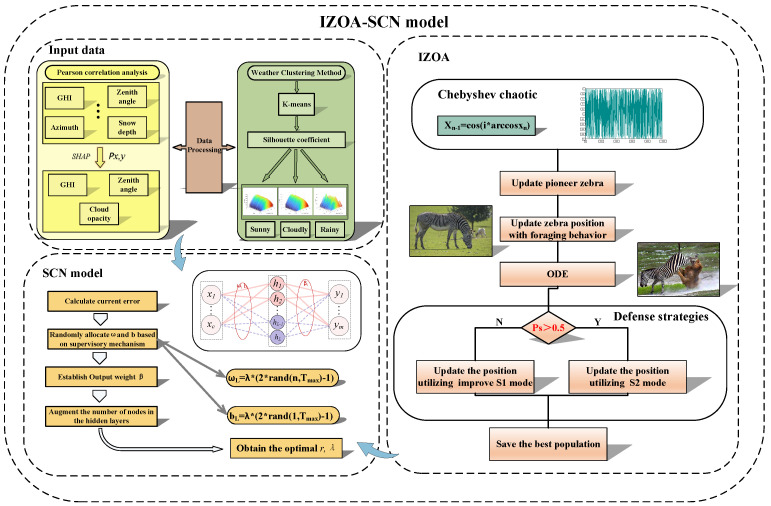
The flowchart of PV power forecasting model.

In summary, the flowchart for PV power forecasting model is as follows (see Figure 6).

## 5. Results and Data Analysis

The experiments are conducted on the MATLAB 2024 platform, utilizing hardware with the following specifications: Intel (R) Core (TM) i7-14650HX 2.20 GHz CPU (Intel Corporation, Santa Clara, CA, USA), 16 GB of RAM, a 64-bit Windows operating system, and an NVIDIA GeForce RTX 4060 graphics card (NVIDIA Corporation, Santa Clara, CA, USA).

The PV power data utilized in this study are collected from a PV power station in the Ningxia, China. PV data is collected at 15 min intervals from March to May 2020, amounting to 3864 points. Since PV power generation produces minimal output at night, the chosen data collection period is from 9:30 a.m. to 8:00 p.m, ensuring that each data point used represents valid PV power generation output. The data for validating the model’s generalizability are obtained from a PV power station in northwest China. The dataset spans from November 2020 to January 2021, with a sampling interval of 15 min. The chosen data collection period is from 9:30 a.m. to 8:00 p.m.

### 5.1. Optimizer Performance Analysis

In this section, to evaluate the correctness of the IZOA algorithm’s improvement strategies and the reliability of selecting the ZOA algorithm, six benchmark test functions are employed to assess the algorithm’s performance. As shown in Table 1, $f_{1}\left(x\right),\;f_{2}\left(x\right),\;and\;f_{3}(x)$ are unimodal test functions, which serve as a good set to evaluating the exploitation capability of the algorithm, since they have only one global optimum and no local optima. $f_{4}\left(x\right),\;f_{5}\left(x\right),\;and\;f_{6}(x)$ are multimodal functions, which provide a suitable test set for assessing the exploration ability of the algorithm, as they contain multiple local optima. As shown in Table 2, the average values (AVG), standard deviations (STD), and optimal values obtained by IZOA, ZOA, PSO, and several classical optimization algorithms on test functions are presented. The AVG is used to demonstrate the central tendency of the algorithms, the STD serves to quantify the dispersion of optimization results, while the optimal values provide an intuitive observation of the algorithms’ solution accuracy. The population size for all algorithms is set to 30, with a maximum iteration count of 500. Each model is independently run 20 times to ensure statistical reliability.

Table 2 demonstrates that, compared to other optimization algorithms on unimodal functions, IZOA achieves solutions that are closest to the theoretical optimum, exhibiting significant performance superiority. This indicates that the improvement strategies proposed in this paper are effective in enhancing solution accuracy. The ZOA algorithm ranks second, obtaining the next-best optimal values after IZOA, which also provides a reliable basis for selecting the ZOA algorithm in this study. Furthermore, IZOA exhibits the lowest STD, indicating superior optimization stability of the model.

In the case of multimodal functions, IZOA still provides relatively accurate optimal solutions for the functions, while ZOA performs second only to IZOA. The simulation results show that both the IZOA and ZOA algorithms possess strong exploration capabilities in precisely scanning the search space and passing local optimal regions.

### 5.2. Data Processing

#### 5.2.1. Meteorological Characteristics Selection

The output power of PV systems is affected by various elements, which are primarily divided into external environmental factors and internal factors of the PV system. Internal factors such as component parameters and the efficiency of PV energy conversion remain relatively stable after the installation of the PV panels and can be ignored during the prediction process. External environmental factors related to weather primarily include GHI, relative humidity, and snow depth, among others.

The paper utilizes Pearson correlation coefficient analysis to select input features closely related to PV power output [36]. Pearson correlation coefficient is calculated by Equation (24).

(24) $$\rho_{x,y}=\frac{Cov\left(X,Y\right)}{\sigma_{x}.\sigma_{y}}=\frac{\sum_{i=1}^{n}\left(x_{i}-E\left(X\right)\right)\left(y_{i}-E\left(Y\right)\right)}{\sqrt{\sum_{i=1}^{n}{\left(X_{i}-E\left(X\right)\right)}^{2}}\cdot \sqrt{\sum_{i=1}^{n}{\left(y_{i}-E\left(Y\right)\right)}^{2}}}$$

Here, Cov(X,Y) is the covariance of variables *X* and *Y*, $\sigma_{x},\sigma_{y}$ is the standard deviations of variables *X* and *Y*. $E\left(X\right),E\left(Y\right)$ is the mathematical expectation of variables *X* and *Y*, and $\rho_{x,y}$ represents the correlation coefficient of variables *X* and *Y*, with a value range of [−1, 1]. The magnitude of the Pearson correlation coefficient indicates the degree of linear relationship between variables *X* and *Y*. A greater absolute value indicates a stronger correlation.

The Pearson correlation coefficients for each input feature with PV output power are presented in Table 3.

According to Table 3, global horizontal irradiance, dew point temperature, and atmospheric precipitation exhibit positive correlations with PV power output. Cloud opacity, zenith angle, azimuth, relative humidity, snow depth, and pressure demonstrate negative correlations with PV power output.

The measured GHI includes direct normal irradiance (DNI) and diffuse horizontal irradiance (DHI). DNI refers to the amount of radiation received when sunlight passes through direct radiation, while DHI is the amount of radiation received after sunlight is scattered by the atmosphere.

The absolute values of the correlation coefficients for GHI with zenith angle are 0.99 and 0.83, demonstrating a strong correlation with PV power output. In addition, the absolute values of the Pearson correlation coefficients with cloud opacity for relative humidity are 0.20 and 0.44, showing a moderate correlation with PV power output. Furthermore, the absolute values of the Pearson correlation on coefficients for other meteorological features are all less than 0.1, showing very weak correlation with PV power output.

This study employs Shapley additive explanation (SHAP) analysis to further enhance the reliability of input feature selection. SHAP is an interpretability framework for machine learning that assigns each feature an importance value reflecting its contribution to the predictions of the model. SHAP values offer a quantitative assessment of feature influence, enabling precise evaluation of how different input variables affect predictive outcomes. SHAP adopts an additive feature attribution method that treats all features as “contributors” jointly influencing the final prediction outcome. This comprehensive approach ensures that each feature’s contribution is quantified relative within all other features. For each individual sample, the model generates a prediction, and the corresponding SHAP values represent the numerical attribution of each feature’s contribution to that specific prediction outcome.

**Figure 7:**
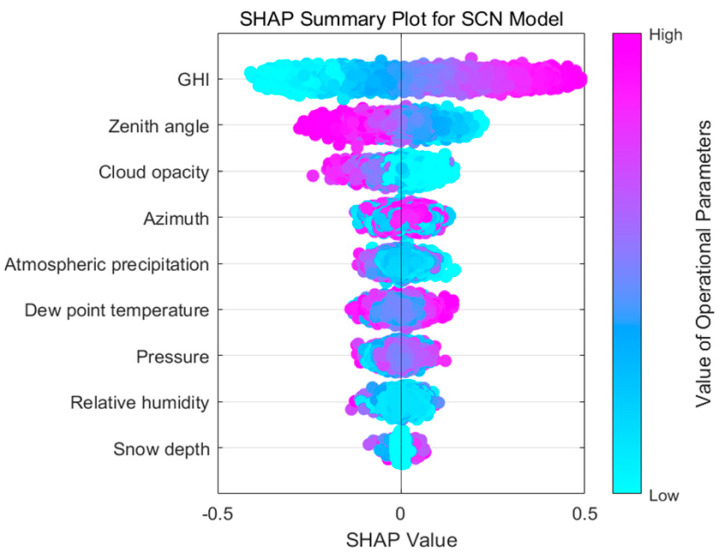
The summary plot for SCN.

**Figure 8:**
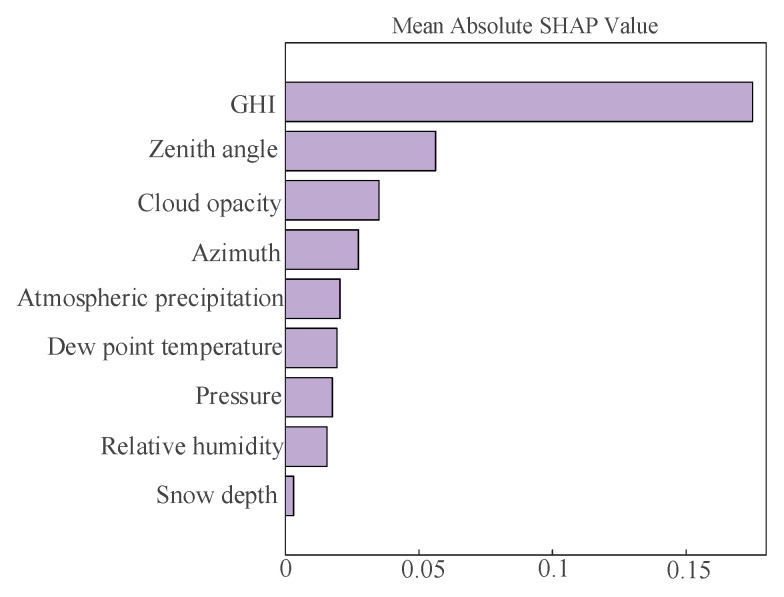
Mean absolute SHAP value.

As shown in Figure 7 and Figure 8, SHAP is employed to interpret the characteristics of input for SCN, with features ranked in descending order of importance based on mean absolute SHAP values. Positive SHAP values correspond to positive effects, while negative values correspond to negative effects. The color bar in Figure 7 illustrates that a color closer to the upper end represents a higher feature value, while a color nearer to the lower end corresponds to a smaller feature value. Furthermore, a wider color bar reflects a more significant feature impact, suggesting that such a feature is more critical.

**Figure 9:**
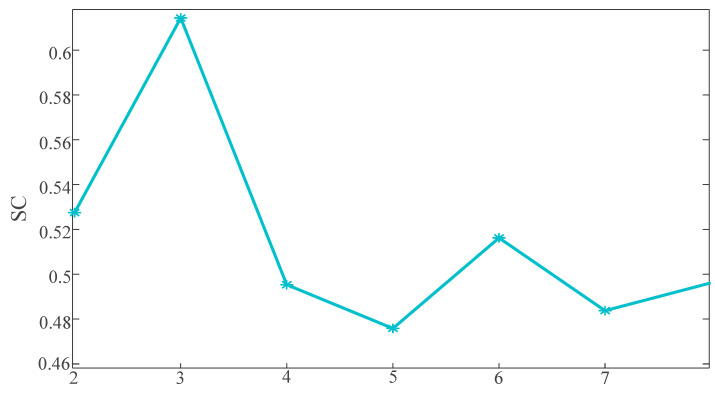
The SC coefficients for different numbers of clusters.

In Figure 7, GHI, zenith angle, and cloud opacity have a higher contribution ranking. GHI exhibits the most significant influence among all variables. The SHAP values of GHI demonstrate a wide distribution. The light purple segments indicate that higher GHI corresponds to greater PV power output, while the light blue segments indicate that lower GHI values correspond to reduced PV power output. Similarly, the zenith angle and cloud opacity also exhibit broad SHAP value distributions. Unlike GHI, their light purple segments correspond to lower PV power output, whereas the pale blue portions are associated with higher PV power output. The contributions of the remaining input features appear less distinct. As shown in Figure 9, GHI, zenith angle, and cloud opacity exhibit the highest mean absolute SHAP values, providing further evidence to support the conclusion derived from Figure 8.

By analyzing both correlation coefficients and SHAP values, this paper chooses GHI, zenith angle, and cloud opacity as input variables for the predictive model.

#### 5.2.2. Weather Clustering Method Based on K-Means

This paper employs K-means clustering to reduce the uncertainty in PV power output caused by weather variations. For two time series sequences X and Y of equal length, each containing *p* samples, the Euclidean distance metric is computed as depicted in Equation (25).

(25) $$D\left(X,Y\right)=\sqrt{\sum_{a=1}^{p}{\left(X_{a}-Y_{a}\right)}^{2}}$$

In Equation (27), *X* and *Y* represent time series sequences of equal length, *D* is Euclidean distance metric.

In K-means clustering, the selection of the number of clusters (K) directly impacts the clustering results. However, the determination of the value of *k* is often defined artificially, lacking a theoretical foundation. Therefore, this paper utilizes the silhouette coefficient (*SC*) to determine the optimal number of clusters. The formula for calculating the *SC* is depicted in Equation (26).

(26) $$SC=\frac{D_{b}-D_{a}}{Max\left(D_{a},D_{b}\right)}$$

Here, $D_{a}$ represents the average distance from a certain sequence to other sequences within the same cluster, and $D_{b}$ denotes the average distance from that sequence to sequences in other clusters. The range of the *SC* coefficient spans from [−1, 1]. The closer SC approaches one, the better the clustering effectiveness. A value of zero indicates cluster overlap.

This paper calculates the SC values for different categories with the number of clusters ranging from two to eight, and the results are illustrated in Figure 9.

**Figure 10:**
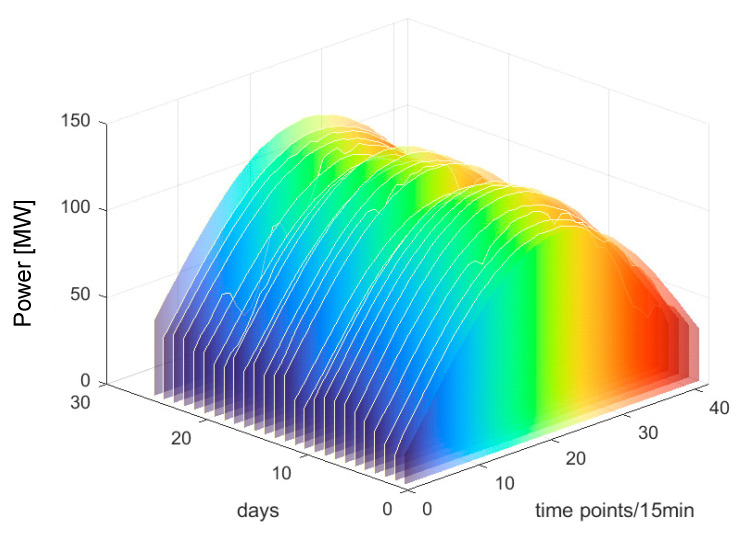
The clustering results of category 1.

**Figure 11:**
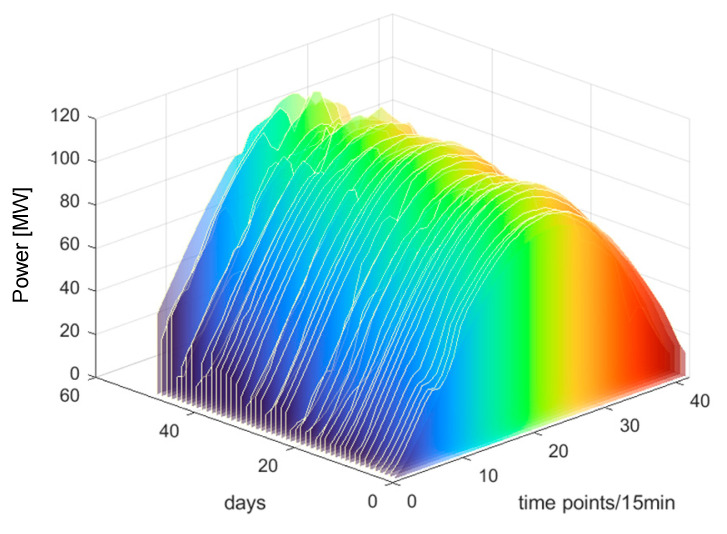
The clustering results of category 2.

**Figure 12:**
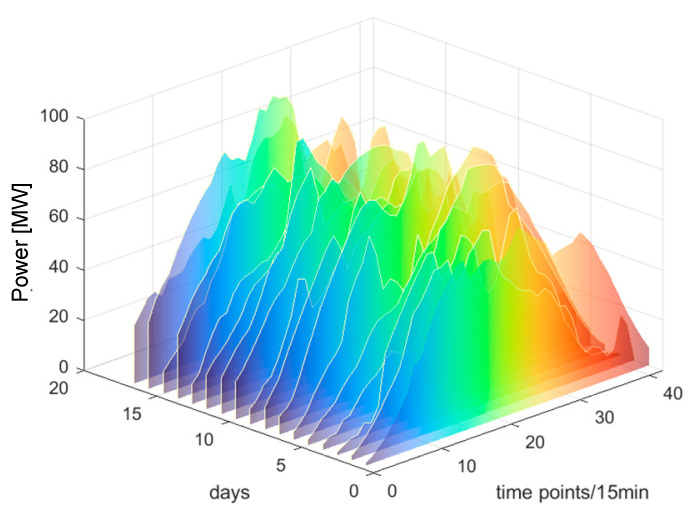
The clustering results of category 3.

Based on Figure 9, it is evident that the SC reaches its peak value of 0.61 when the number of clusters is set to three. Therefore, this paper chooses three as the number of clusters. The clustering outcomes are illustrated in Figure 10, Figure 11 and Figure 12.

From Figure 10, Figure 11 and Figure 12, it can be observed that each PV power curve of various categories exhibits unique characteristics [37]. Among the three types of photovoltaic (PV) output curves, category 1 has the highest peak. Its peaks are generally above 100 MW and exhibit obvious regularity. The occurrence of this situation can be attributed to the consistent sunlight radiation received by the PV panels, thereby resulting in no fluctuations in PV power. Therefore, category 1 is defined as sunny patterns. The peak values of category 2 curves are typically below 100 MW, primarily due to the intermittent shading of solar panels caused by the movement of cloud. Therefore, category 2 is defined as cloudy patterns. Category 3 curves are the most special, featuring the lowest peak and exhibiting marked volatility. This is due to the fact that the solar radiation received by PV panels becomes unstable on rainy days or sudden weather changes. Category 3 is defined as rainy patterns. This study establishes short-term PV power prediction model under the aforementioned three weather patterns.

### 5.3. Results

#### 5.3.1. Evaluating Indicator

The paper utilizes the mean absolute error (*MAE*), mean square error (*MSE*), root mean square error (*RMSE*), mean absolute percentage error (*MAPE*), and the coefficient of determination (*R*^2^) to evaluate the predictive model performance [38], with specific formulas as follows:

(27) $$MAE=\frac{1}{n}\sum_{i=1}^{n}\left|y_{i}-y_{i}^{*}\right|$$

(28) $$MSE=\frac{1}{n}\sum_{i=1}^{n}{\left(y_{i}-y_{i}^{*}\right)}^{2}$$

(29) $$RMSE=\sqrt{\frac{1}{n}\sum_{i=1}^{n}{\left(y_{i}-y_{i}^{*}\right)}^{2}}$$

(30) $$MAPE=\frac{1}{n}\sum_{i}^{n}\frac{\left|y_{i}-y_{i}^{*}\right|}{y_{i}}$$

(31) $$R^{2}=1-\frac{\sum_{i=1}^{n}{\left(y_{i}-y_{i}^{*}\right)}^{2}}{\sum_{i=1}^{n}\left(y_{i}-\bar{y}\right)}$$

where $y_{i}$ denotes the true value of the PV power for the *i*-th sample, $y_{i}^{*}$ represents the predicted value of the PV power for the *i*-th sample, *n* signifies the number of samples, and $\bar{y}$ indicates the average PV power.

#### 5.3.2. Forecast Results and Analysis

The parameter settings for the IZOA-SCN are as follows: population size (pop) is 30, the maximum iterations $\left(G_{max}\right)$ is 50, population dimensions (dim) is 2, maximum number of candidate nodes ($T_{max})$ is 100, tolerance error (ε) is 0.01, the maximum number of the hidden nodes $\left(L_{max}\right)$ is 50, and the activation function g(x) is sigmoid. The upper bound ($u_{b}$) for r is set at 0.9, and the lower bound ($l_{b}$) is set at 0.9999999. The upper bound ($u_{b}$) for λ is set at 250, and the lower bound ($l_{b}$) is set at 0.5 [39].

**Figure 13:**
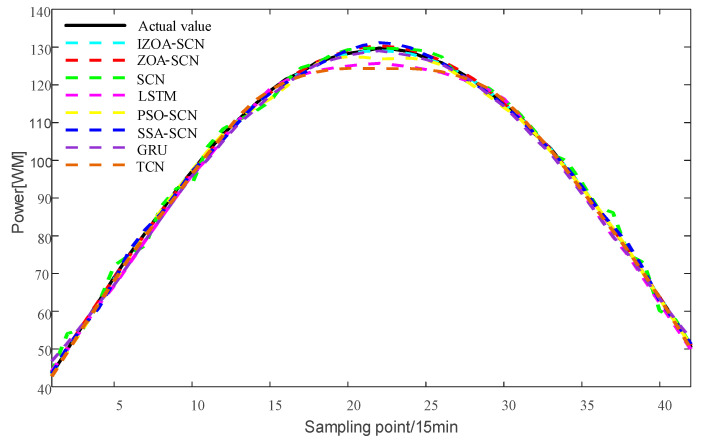
The predicted power output curves on sunny pattern.

**Figure 14:**
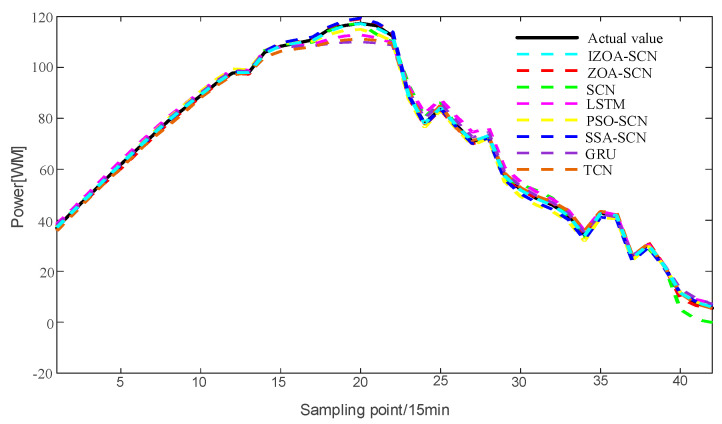
The predicted power output curves on cloudy pattern.

**Figure 15:**
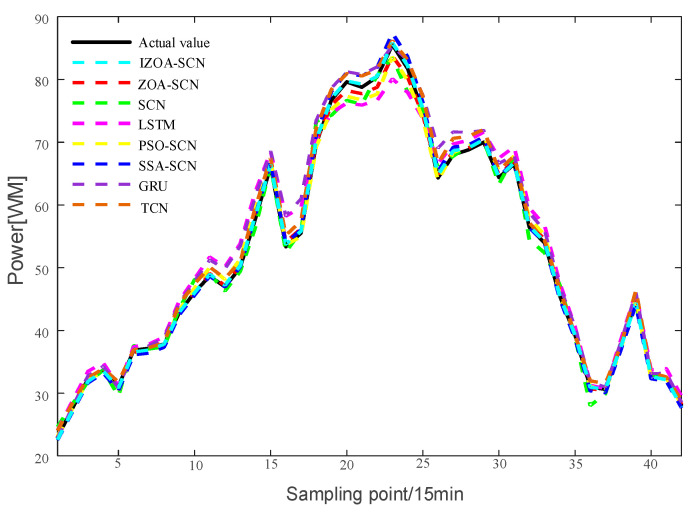
The predicted power output curves on rainy pattern.

**Figure 16:**
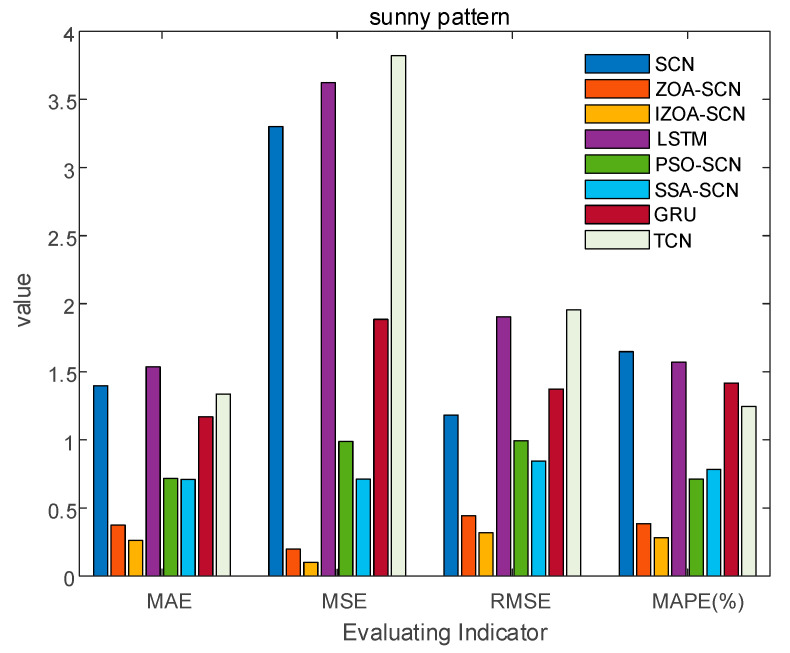
The bar chart of sunny pattern evaluation indicator.

**Figure 17:**
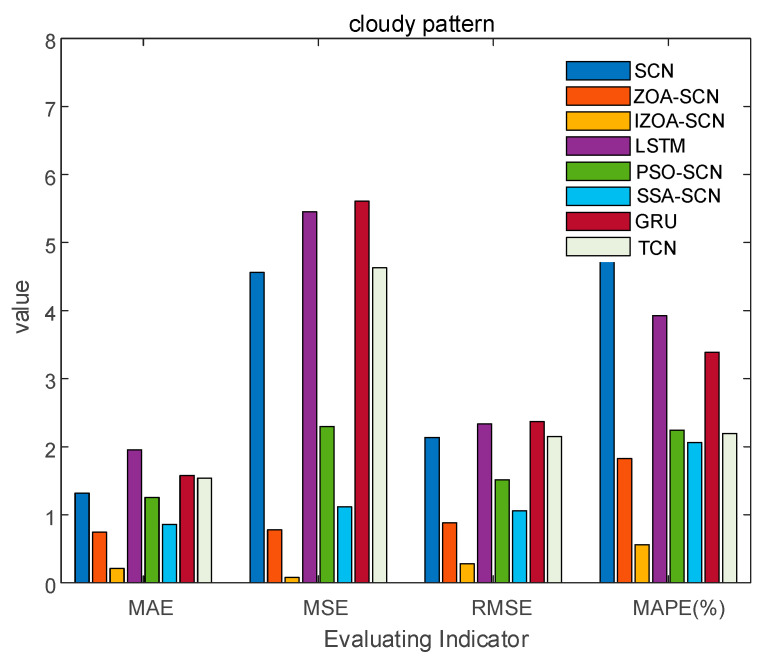
The bar chart of cloudy pattern evaluation indicator.

**Figure 18:**
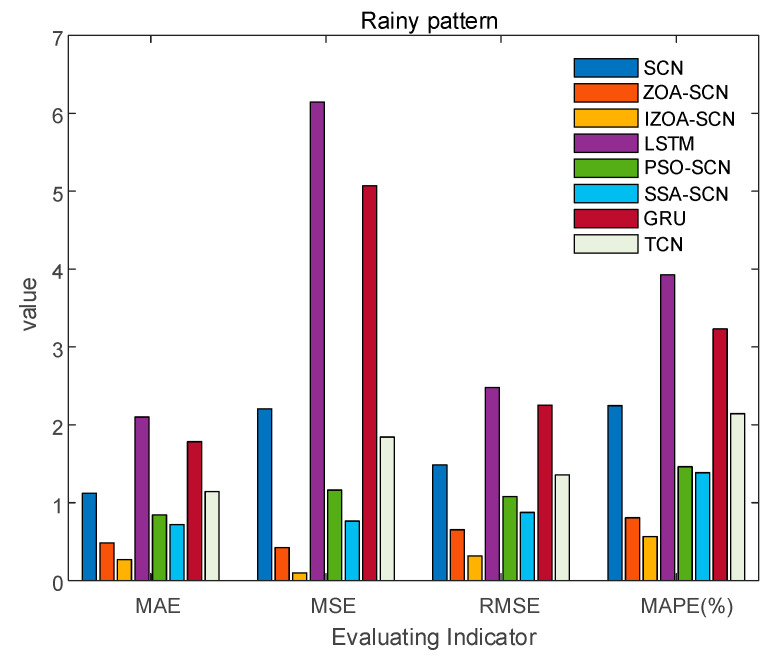
The bar chart of rainy pattern evaluation indicator.

To verify the accuracy of the proposed model, the paper compares the predictive results of SCN, ZOA-SCN, LSTM, PSO-SCN, SSA-SCN, TCN, and GRU. Each model is run independently 100 times to record the optimal results. The predicted photovoltaic power generation under different weather patterns is compared using five assessment indicators, further validating the precision of each prediction model. The prediction results are illustrated for the sunny pattern in Figure 13, for the cloudy pattern in Figure 14, and for the rainy pattern in Figure 15. The evaluation indicator is presented in Table 2, Table 3 and Table 4. The bar charts shown in Figure 16, Figure 17 and Figure 18 display the evaluation metrics for various weather patterns.

In the case of the sunny pattern, Figure 13 demonstrates the forecasting results from various models. The prediction curves of SCN, LSTM, and GRU exhibit similar trends to the actual PV power curve. However, GRU and LSTM predict values that are significantly lower than the actual value near the peak. The SCN predicts values that are higher than the true values near the peak, with slight fluctuations occurring at other time points. Thanks to the architecture of the SCN network, the fitting performance of the SCN network is generally superior to that of the LSTM and GRU overall. The performance of SCN in predicting around the peak is unstable due to the influence of manually set hyperparameters on the SCN. ZOA-SCN is capable of selecting the optimal values for SCN model parameters, thereby diminishing model error. As illustrated by the red curve in Figure 13, the fitting curve of ZOA-SCN is essentially consistent with the true values. The tracking performance of PSO-SCN and SSA-SCN in the peak region is second only to that of ZOA-SCN. However, traditional ZOA is prone to falling into local optima, which restricts the precision of the model and produces deviations from the true values at later points of prediction. The IZOA-SCN method can effectively predict PV power under sunny pattern, showing superior generalization performance and robustness.

To enhance the understanding of the prediction results, this study adopted a more intuitive bar chart to present the evaluation indicators, as shown in Figure 14 and Table 4, which displays the distribution of evaluation metrics for the four models under sunny pattern. It can be intuitively seen that, due to the unique supervision mechanism of the SCN, the performance indices of the SCN are generally superior to the LSTM. Additionally, the method of optimizing the parameters of the SCN through ZOA and IZOA has significantly improved the prediction performance. Compared to SCN, ZOA-SCN reduced the MAE, MSE, RMSE, and MAPE by 73.3%, 92.1%, 75.6%, and 76.7%, respectively. The IZOA proposed in this paper effectively addresses the issue of ZOA being prone to get stuck in local optima and being unable to generate the optimal parameter set. IZOA-SCN performed even better in all aspects. Compared to ZOA-SCN, IZOA-SCN reduced the MAE, MSE, RMSE, and MAPE by 30.2%, 61.2%, 28.4%, and 26.4%, respectively. The above analysis indicates that the proposed prediction model in this paper has the highest accuracy under sunny pattern.

Regarding the cloudy pattern, the actual curve has some fluctuations. Figure 15 shows the prediction results of IZOA-SCN, ZOA-SCN, SCN, PSO-SCN, SSA-SCN, GRU, TCN, and LSTM. Obviously, the LSTM and GRU model has the worst fitting performance. After the actual values start to fluctuate, LSTM and GRU fail to effectively capture the changes in the actual PV power curve and gradually deviate from the real values. Although SCN can capture overall trends, it still exhibits errors at some turning points, failing to effectively capture local features, especially after the 25th sampling point. ZOA-SCN demonstrates excellent local fitting ability after adopting an optimized algorithm to obtain better parameter sets. IZOA-SCN demonstrates the best fitting effect due to the introduction of the improvement strategy to further enhance the ability of ZOA to search parameters.

Based on Figure 16 and Table 5, it can be observed that the MSE and MAPE of the models are relatively high. This indicates that the significant difference in waveform between the previous and subsequent periods under cloudy pattern, making prediction challenging. However, the evaluation indicators of models based on SCN are generally superior to those of LSTM, which also confirms the superiority of SCN. The ability of SCN to randomly allocate parameters is influenced by the parameter range of artificial settings, which prevents SCN from achieving the optimal prediction effect. Due to the combination of ZOA, the performance indicators of ZOA-SCN have been significantly improved. Compared to SCN, the MAE, MSE, RMSE, and MAPE of ZOA-SCN decreased by 43.6%, 82.9%, 58.7%, and 70.1%, respectively. Compared to ZOA-SCN, the MAE, MSE, RMSE, and MAPE of the IZOA-SCN are further decreased by 71.7%, 90.0%, 68.4%, and 69.3%, respectively. Considering the analysis provided above, the IZOA-SCN model proposed in this paper has the best evaluation indicator, once again verifying the accuracy of the IZOA-SCN, and demonstrating its superior predictive performance in situations of greater volatility.

Concerning the rainy pattern, owing to the impact of weather changes, the actual PV power curve exhibits multiple fluctuations. Figure 17 shows the prediction results of IZOA-SCN, ZOA-SCN, SCN, PSO-SCN, SSA-SCN, GRU, TCN, and LSTM. As shown in Figure 17, before the 15th sampling point, LSTM and SCN produced different degrees of prediction errors. However, as the network deepens, they gradually become able to capture the fluctuations in actual PV power. Despite ZOA-SCN being able to track the trends of the actual PV power curve largely, there remains a potential issue of overfitting. IZOA-SCN effectively addressed this problem, maintaining the best prediction results even during multiple fluctuations.

The Figure 18 and Table 6 show that both SCN and LSTM have poor evaluation indicators. With the automatic optimization of the parameters for SCN, both the MAE and RMSE of ZOA-SCN and IZOA-SCN are less than one, but the MAPE of ZOA-SCN is relatively high. After implementing three improvement strategies on ZOA, the MAPE of IZOA-SCN decreased to 0.5656, while MAE, MSE, and RMSE decreased to 0.2709, 0.0993, and 0.3152, respectively. Additionally, the coefficient of determination reached 0.99998, achieving the best evaluation metrics and effectively improving the accuracy of predictions.

**Figure 19:**
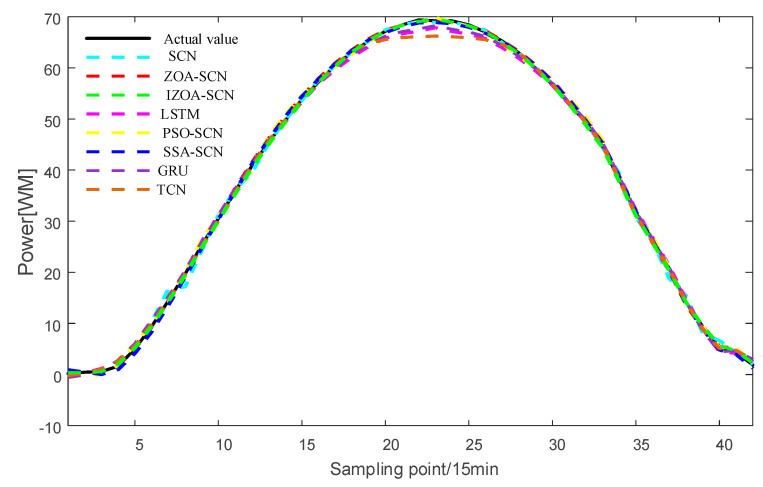
The predicted power output curves on sunny pattern.

**Figure 20:**
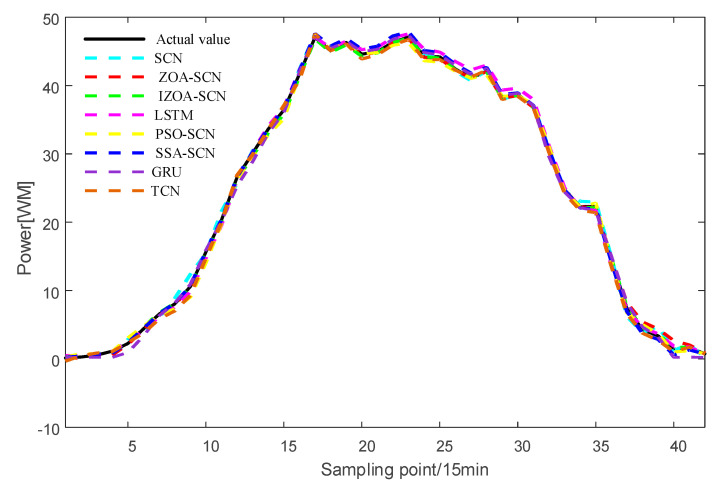
The predicted power output curves on cloudy pattern.

**Figure 21:**
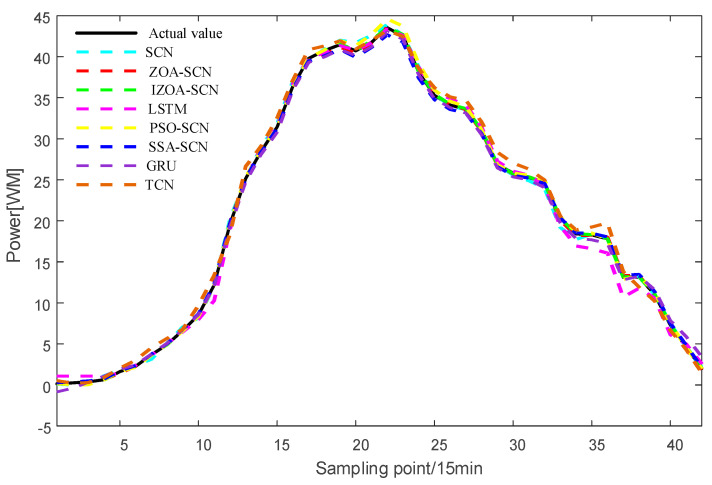
The predicted power output curves on rainy pattern.

To enhance the generalization capability of the model, this study further validates the proposed IZOA-SCN algorithm using a dataset from a PV power station in northwest China [40]. After data processing and clustering, three distinct PV output patterns corresponding to different weather are identified: sunny, cloudy, and rainy patterns. The prediction results are presented in Figure 19, Figure 20 and Figure 21, with the corresponding evaluation indicators shown in Table 5, Table 6 and Table 7.

As shown in Figure 19 and Table 7, under sunny pattern, the LSTM, GRU, and TCN models all exhibit significant deviations in the peak power segment, while the SCN-based model demonstrates closer alignment with the actual values. The SSA-SCN and PSO-SCN models show improved prediction accuracy due to the incorporation of optimization algorithms but still exhibit fluctuations at certain points. In contrast, the ZOA-SCN model successfully overcomes limitations. Among all models, IZOA-SCN demonstrates the best fitting performance, which is further corroborated by its corresponding lowest evaluation indicator. As illustrated in Figure 20 and Figure 21 and Table 8 and Table 9, the models’ tracking capabilities face challenges under cloudy and rainy pattern, where frequent weather variations cause multi-point fluctuations in the actual values. Under these demanding conditions, the proposed IZOA-SCN maintains superior tracking performance and the lowest evaluation indicator among all compared methods. Therefore, the comprehensive simulation results conclusively demonstrate that IZOA-SCN effectively enhances the accuracy of short-term photovoltaic power forecasting.

According to Table 10, due to its unique network architecture, the SCN achieves the shortest runtime while possessing accurate prediction results compared to LSTM, TCN, and GRU. The PSO-SCN, SSA-SCN, and ZOA-SCN models exhibit increased computational time compared to the original SCN due to the introduction of optimization algorithms. However, they still outperform recurrent neural networks such as LSTM and GRU in terms of efficiency. Although IZOA-SCN has a relatively elapsed time, the above simulation experiments demonstrate that its prediction accuracy is significantly improved, thereby compensating for the temporal drawback. This demonstrates the feasibility of the proposed IZOA-SCN model for short-term PV power forecasting. An important future work of this research is to optimize the network architecture to reduce elapsed time while maintaining prediction accuracy.

#### 5.3.3. Model Evaluation Based on Statistical Experiments

In model evaluation, relying on indicators such as MAE and MSE to compare the advantages of different models is not entirely reliable. Therefore, this study further employs Friedman and Wilcoxon tests to demonstrate the efficacy of the IZOA-SCN algorithm.

The Friedman test is a non-parametric statistical method used to compare whether a group of algorithms exhibits statistically significant differences. It evaluates the null hypothesis that “all algorithms perform equally” by comparing the resulting *p*-value. If the *p*-value is less than the significance level (set at 0.05 in this study), the null hypothesis is rejected, indicating that “the algorithms’ performances differ significantly”. Conversely, if the *p*-value exceeds the significance level, the null hypothesis cannot be rejected. This study compares the *p*-values of seven different algorithms, with the detailed results presented in Table 11, Table 12 and Table 13.

As shown in Table 11, Table 12 and Table 13, the *p*-values of Friedman test are below 0.05 across all weather patterns, indicating statistically significant differences among at least two models. Subsequent Wilcoxon signed-rank tests further demonstrate that the proposed IZOA-SCN algorithm exhibits significant performance differences compared to all algorithms. Therefore, when combined with evaluation indicators, these statistical results confirm IZOA-SCN as the optimal algorithm.

#### 5.3.4. Model Ablation Study

**Figure 22:**
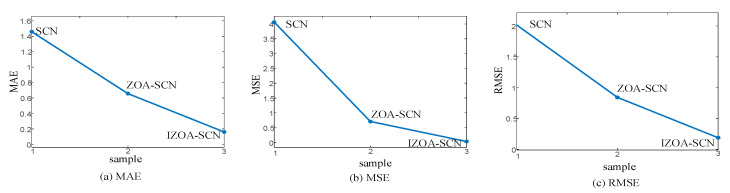
The comparison chart of ablation experiment indicator across.

In this section, a series of ablation experiments will be conducted to demonstrate that each component of the proposed IZOA-SCN algorithm has a significant impact on PV power prediction results. Figure 22 and Table 14 present the ablation analysis results of the MAE, MSE, and RMSE for one-day PV power prediction.

The results demonstrate that both the ZOA optimization algorithm and its improvement strategies are essential for achieving high accuracy and low error, as they can effectively collaborate with each other. When the ZOA algorithm component is removed, the MAE, MSE, and RMSE decreased by 54.9%, 82.5%, and 58.2%, respectively, compared to the SCN model. This indicates that the ZOA algorithm effectively assists the SCN in obtaining optimal values for parameters, thereby significantly improving prediction accuracy. When the IZOA algorithm component is removed, the MAE, MSE, and RMSE increased by 88.97%, 99.11%, and 90.56%, respectively, compared to the SCN model. This demonstrates that IZOA plays a pivotal role in minimizing prediction errors to the greatest extent. Moreover, when the improvement component of ZOA is removed, the MAE, MSE, and RMSE increased by 75.56%, 94.91%, and 77.3%, respectively, compared to the complete IZOA-SCN model. This clearly demonstrates that the proposed IZOA algorithm effectively addresses the drawbacks of the original ZOA method. In summary, the ablation experiments conclusively validate the effectiveness of the proposed method. The results demonstrate that the integrated approach achieves optimal performance in PV power prediction when all components are implemented, fully realizing its advantages.

The proposed model in this paper involves a small number of manually set parameters. To make these parameter settings more reliable, ablation experiments are conducted on the parameters. First, in the SCN network architecture, while keeping $T_{max}$ constant, the number of $L_{max}$ is varied to 25, 50, 100, 125, and 150, with the results shown in the Table 15. Next, while keeping $L_{max}$ constant, the number of $T_{max}$ is adjusted to 25, 50, 75, 100, and 125, with the results presented in the Table 16.

According to Table 15 and Table 16, the study observes that when the maximum number of hidden nodes is fixed, the MAE, MSE, and RMSE of the SCN network architecture decrease sharply as $L_{max}$ increases, until $L_{max}$ reaches 100. However, further increasing $L_{max}$ beyond this point leads to a gradual rise in MAE, MSE, and RMSE. On the other hand, when $L_{max}$ is fixed, increasing $T_{max}$ beyond 50 results in diminishing returns, as the model fails to capture features more effectively. These analyses indicate that, for the based SCN architecture, the optimal parameter combination is $T_{max}$ = 50 and $L_{max}$ = 100, at which point the model achieves its best feature extraction capability.

For the ZOA-SCN model, the population size (*pop*) and the maximum number of iterations $\left(G_{max}\right)$ significantly influence the optimization capability of the ZOA. First, we keep the *pop* constant and varied $G_{max}$ to 40, 45, 50, 55, and 60, with the results shown in the Table 17. Next, we fixed $G_{max}$ and adjusted the *pop* to 20, 25, 30, 35, and 40, with the results presented in Table 18.

We observed that increasing either $G_{max}$ or *pop* may prevent ZOA from converging to the optimal value when solving optimization. Excessively high *pop* and $G_{max}$ may lead to model overfitting, while setting these parameters too low could prevent the model from searching for high-quality solutions in ZOA. When $G_{max}$ = 50 and *pop* = 30, ZOA enables SCN to achieve the best parameters, resulting in the smallest MAE, MSE, and RMSE.

## 6. Conclusions

In the context of global environmental degradation, the output of PV power generation is significantly influenced by weather changes, presenting a high degree of uncertainty. The precision of PV power prediction has presented as a notable challenge. Applying K-means clustering for classifying historical PV data significantly reduces the uncertainty caused by environmental factors on the output of PV power. Moreover, this study proposes an effective short-term PV power forecasting model based on IZOA-SCN. With three improvement strategies implemented for ZOA, the IZOA algorithm is provided for selecting the regularization parameters and scale factors of SCN, effectively addressing the issue of unstable prediction results due to manual setting of hyperparameters. Compared to LSTM, SCN, and ZOA-SCN, the proposed model exhibits the best prediction effect under three types of weather patterns, demonstrating superior generalization capabilities and robustness.

It is noteworthy that, with the progress of SCN research, robust SCN and deep SCN have been proposed, which may improve prediction performance. This study will attempt to replace the foundational model with robust SCN and deep SCN in the future, aiming to further explore the application of SCN in the field of PV power prediction.

The IZOA-SCN prediction model proposed in this study can accurately predict photovoltaic (PV) power under three common weather conditions. Accurate PV power prediction can effectively reduce solar curtailment. Beyond the field of PV power generation, the proposed model can also be applied to the prediction of other renewable energy sources, contributing to the advancement of renewable energy development. However, the model still has limitations. Due to the lack of consideration for certain extreme weather conditions, which exhibit more complex and harder-to-capture characteristics, the relationship between these conditions and PV power output becomes more intricate. Therefore, future research will focus on PV power prediction under such extreme weather conditions to enhance the robustness of models.

## Figures and Tables

**Table 1:** Test function.

Function	Dimension	Range
$f_{1}\left(x\right)=\sum_{i=1}^{30}x_{i}^{2}$	30	[−100, 100]
$f_{2}\left(x\right)=\sum_{i=1}^{30}\left|x_{i}\right|+\prod_{i=1}^{30}\left|x_{i}\right|$	30	[−10, 10]
$f_{3}\left(x\right)=\sum_{i=1}^{30}{\left(\sum_{j=1}^{i}x_{j}\right)}^{2}$	30	[−100, 100]
$f_{4}\left(x\right)=\sum_{i=1}^{30}\left[x_{i}^{2}-10cos\left(2\pi x_{i}\right)+10\right]$	30	[−5.12, 5.12]
$f_{5}\left(x\right)=-20exp\left(-0.2\sqrt{\frac{1}{30}\sum_{i=1}^{30}x_{i}^{2}}\right)-exp\left(\frac{1}{30}\sum_{i=1}^{30}cos\left(2\pi x_{i}\right)\right)+20+e$	30	[−32, 32]
$f_{6}\left(x\right)=\sum_{i=1}^{11}{\left[a_{i}-\frac{x_{1}\left(b_{i}^{2}+b_{i}x_{2}\right)}{b_{i}^{2}+b_{i}x_{3}+x_{4}}\right]}^{2}$	30	[−5, 5]

**Table 2:** Test results.

Function	Algorithms	AVG	STD	Optimal
$f_{1}\left(x\right)$	IZOA	7.0024 × 10^−314^	0	4.9407 × 10^−323^
	ZOA	4.9393 × 10^−251^	0	2.3053 × 10^−261^
	GWO	4.2078 × 10^−27^	1.2424 × 10^−26^	5.8831 × 10^−29^
	WOA	5.4801 × 10^−75^	1.7245 × 10^−74^	2.4443 × 10^−83^
	PSO	0.25409	0.24676	0.079196
	RIME	2.0523	0.67872	1.2152
$f_{2}\left(x\right)$	IZOA	6.1121 × 10^−171^	0	3.8899 × 10^−175^
	ZOA	2.922 × 10^−132^	7.7955 × 10^−132^	2.6854 × 10^−138^
	GWO	8.4728 × 10^−17^	5.5396 × 10^−17^	1.2437 × 10^−17^
	WOA	2.4134 × 10^−52^	7.2024 × 10^−52^	1.1527 × 10^−56^
	PSO	0.063561	0.024854	0.032937
	RIME	1.4435	1.0873	0.4416
$f_{3}\left(x\right)$	IZOA	4.9213 × 10^−190^	0	7.8331 × 10^−214^
	ZOA	7.7994 × 10^−161^	2.4663 × 10^−160^	3.9772 × 10^−180^
	GWO	9.9272 × 10^−6^	2.7574 × 10^−5^	2.8674 × 10^−8^
	WOA	38288.916	14914.0565	11776.3969
	PSO	2384.4845	2198.1302	606.7415
	RIME	1320.7304	415.1079	611.877
$f_{4}\left(x\right)$	IZOA	0	0	0
	ZOA	0	0	0
	GWO	1.5218	2.7839	0
	WOA	5.6843 × 10^−15^	1.7975 × 10^−14^	0
	PSO	49.8859	9.6766	34.4649
	RIME	56.1388	15.7847	32.1465
$f_{5}\left(x\right)$	IZOA	4.4409 × 10^−16^	0	4.4409 × 10^−16^
	ZOA	4.4409 × 10^−16^	0	4.4409 × 10^−16^
	GWO	1.0134 × 10^−13^	1.9036×10^−14^	7.8604 × 10^−14^
	WOA	4.7073 × 10^−15^	2.2469×10^−15^	4.4409 × 10^−16^
	PSO	0.46163	0.47346	0.084786
	RIME	2.2076	0.29868	1.891
$f_{6}\left(x\right)$	IZOA	7.0024 × 10^−314^	0	4.9407 × 10^−323^
	ZOA	4.9393 × 10^−251^	0	2.3053 × 10^−261^
	GWO	4.2078 × 10^−27^	1.2424 × 10^−26^	5.8831 × 10^−29^
	WOA	5.4801 × 10^−75^	1.7245 × 10^−74^	2.4443 × 10^−83^
	PSO	0.25409	0.24676	0.079196
	RIME	2.0523	0.67872	1.2152

**Table 3:** Correlation coefficient between input features and PV power.

Attributes	$\rho_{x,y}$
Global horizontal irradiance	0.99
Cloud opacity	−0.20
Zenith angle	−0.83
Azimuth	−0.03
Dew point temperature	0.06
Atmospheric precipitation	0.07
Relative humidity	−0.44
Snow depth	−0.08
Pressure	−0.05

**Table 4:** Sunny pattern evaluation indicator.

Model (Sunny)	MAE	MSE	RMSE	MAPE (%)	$R^{2}$
SCN	1.3984	3.3014	1.8170	1.6487	0.9977
ZOA-SCN	0.3739	0.2609	0.4441	0.3836	0.9998
IZOA-SCN	0.2609	0.1011	0.3180	0.2824	0.9999
LSTM	1.5364	3.6232	1.9035	1.5708	0.9988
PSO-SCN	0.7174	0.9882	0.9941	0.7122	0.9996
SSA-SCN	0.7095	0.7110	0.8438	0.7837	0.9994
TCN	1.3371	3.8214	1.9548	1.2451	0.9983
GRU	1.1699	1.8855	1.3731	1.4182	0.9992

**Table 5:** Cloudy pattern evaluation indicator.

Model (Cloudy)	MAE	MSE	RMSE	MAPE (%)	$R^{2}$
SCN	1.3165	4.5598	2.1354	6.2283	0.9982
ZOA-SCN	0.7431	0.7779	0.8820	1.8263	0.9991
IZOA-SCN	0.2101	0.0779	0.2791	0.5601	0.9997
LSTM	1.9525	5.4550	2.3356	3.9253	0.9981
PSO-SCN	1.2554	2.2969	1.5155	2.2459	0.9991
SSA-SCN	0.8594	1.1176	1.0572	2.0612	0.9995
TCN	1.538	4.6289	2.1515	2.1954	0.9988
GRU	1.5760	5.6070	2.3679	3.3880	0.9980

**Table 6:** Rainy pattern evaluation indicator.

Model (Rainy)	MAE	MSE	RMSE	MAPE (%)	$R^{2}$
SCN	1.1213	2.2044	1.4847	2.2454	0.9968
ZOA-SCN	0.4832	0.4249	0.6519	0.8074	0.9994
IZOA-SCN	0.2709	0.0993	0.3152	0.5656	0.9998
LSTM	2.0995	6.1442	2.4788	3.9241	0.9927
PSO-SCN	0.8419	1.1625	1.0782	1.4624	0.9983
SSA-SCN	0.7193	0.7652	0.8747	1.3840	0.9990
TCN	1.1436	1.8426	1.3574	2.1431	0.9995
GRU	1.7836	5.0694	2.2515	3.2302	0.9977

**Table 7:** Sunny pattern evaluation indicator.

Model (Sunny)	MAE	MSE	RMSE	MAPE (%)	$R^{2}$
SCN	0.5165	0.6264	0.7914	6.8209	0.9995
ZOA-SCN	0.3685	0.1967	0.4434	1.8358	0.9998
IZOA-SCN	0.2432	0.0836	0.2892	4.6897	0.9999
LSTM	0.7193	0.7880	0.8877	12.5643	0.9990
PSO-SCN	0.4795	0.3583	0.5986	6.6481	0.9997
SSA-SCN	0.4608	0.2662	0.5159	15.4201	0.9998
GRU	0.6156	0.5710	0.7756	18.3660	0.9994
TCN	0.6473	1.2211	1.1050	12.5053	0.9994

**Table 8:** Cloudy pattern evaluation indicator.

Model (Cloudy)	MAE	MSE	RMSE	MAPE (%)	$R^{2}$
SCN	0.4579	0.3647	0.6039	12.2580	0.9995
ZOA-SCN	0.2338	0.1389	0.3728	9.0799	0.9998
IZOA-SCN	0.1888	0.0537	0.2319	6.1443	0.9999
LSTM	0.5144	0.3706	0.6087	12.9821	0.9994
PSO-SCN	0.4451	0.3414	0.5843	12.0781	0.9995
SSA-SCN	0.3619	0.2075	0.4555	8.1989	0.9997
GRU	0.4729	0.3563	0.5969	14.4412	0.9995
TCN	0.4392	0.2934	0.5416	14.5031	0.9995

**Table 9:** Rainy pattern evaluation indicator.

Model (Rainy)	MAE	MSE	RMSE	MAPE (%)	$R^{2}$
SCN	0.3099	0.1791	0.4232	7.0937	0.9996
ZOA-SCN	0.1427	0.0314	0.1772	6.4546	0.9998
IZOA-SCN	0.0512	0.0053	0.0732	0.7495	0.9999
LSTM	0.5841	0.6699	0.8184	16.4726	0.9985
PSO-SCN	0.2644	0.1645	0.4056	6.5522	0.9998
SSA-SCN	0.2789	0.1294	0.3598	6.4320	0.9998
GRU	0.4664	0.2962	0.5442	16.3502	0.9995
TCN	0.7261	0.7188	0.8478	15.955	0.9989

**Table 10:** Elapsed time of the model.

Weather Pattern	Model	Elapsed Time (s)
Sunny	SCN	1.779
	ZOA-SCN	13.871
	IZOA-SCN	30.652
	LSTM	20.173
	PSO-SCN	14.527
	SSA-SCN	17.769
	GRU	18.449
	TCN	15.592
Cloudy	SCN	1.850
	ZOA-SCN	16.987
	IZOA-SCN	38.551
	LSTM	21.024
	PSO-SCN	18.618
	SSA-SCN	17.301
	GRU	20.673
	TCN	17.099
Rainy	SCN	1.971
	ZOA-SCN	20.745
	IZOA-SCN	45.501
	LSTM	20.767
	PSO-SCN	22.219
	SSA-SCN	23.444
	GRU	21.394
	TCN	20.915

**Table 11:** Statistical comparison results under sunny pattern.

Model (Sunny)	Wilcoxon Text	Friedman Text
ZOA	0.011342 < 0.05	
SCN	0.0067885 < 0.05	
LSTM	1.9587 × 10^−6^ < 0.05	
SSA-SCN	0.022494 < 0.05	5.223610314 × 10^−15^ < 0.05
PSO-SCN	7.8198 × 10^−6^ < 0.05	
GRU	0.00010889 < 0.05	
TCN	9.3297 × 10^−5^ < 0.05	

**Table 12:** Statistical comparison results under cloudy pattern.

Model (Cloudy)	Wilcoxon Text	Friedman Text
ZOA	0.019704 < 0.05	
SCN	0.0012284 < 0.05	
LSTM	0.0048087 < 0.05	
SSA-SCN	0.0062941 < 0.05	1.1907238022 × 10^−10^ < 0.05
PSO-SCN	0.014011 < 0.05	
GRU	0.0048087 < 0.05	
TCN	0.0060593 < 0.05	

**Table 13:** Statistical comparison results under rainy pattern.

Model (Rainy)	Wilcoxon Text	Friedman Text
ZOA	1.8409 × 10^−6^ < 0.05	
SCN	0.013531 < 0.05	
LSTM	0.0067885 < 0.05	
SSA-SCN	9.3297 × 10^−5^ < 0.05	1.01269394479 × 10^−30^ < 0.05
PSO-SCN	1.6479 × 10^−8^ < 0.05	
GRU	2.5418 × 10^−8^ < 0.05	
TCN	1.6479 × 10^−8^ < 0.05	

**Table 14:** Comparison of ablation experiment indicator.

Model	MAE	MSE	RMSE
IZOA-SCN	0.1608	0.0361	0.1901
ZOA-SCN	0.6580	0.7087	0.8418
SCN	1.4587	4.0571	2.0142

**Table 15:** Ablation study on hidden nodes.

Hidden Nodes	$\mathrm{MAE}\;(T_{max}=50$)	$\mathrm{MSE}\;(T_{max}=50)$	$\mathrm{RMSE}\;(T_{max}=50$)
$L_{max}=25$	3.3908	19.0944	4.3697
$L_{max}=50$	2.265	8.7172	2.9525
$L_{max}=75$	2.1474	7.9684	2.8228
$L_{max}=100$	1.4637	4.1835	2.0454
$L_{max}=125$	2.1468	8.0745	2.8416
$L_{max}=150$	2.1879	8.4540	2.9076

**Table 16:** Ablation study on candidate nodes.

Candidate Nodes	$\mathrm{MAE}\;(L_{max}=100$)	$\mathrm{MSE}\;(L_{max}=100)$	$\mathrm{RMSE}\;(L_{max}=100$)
$T_{max}=25$	2.7614	17.9493	4.2367
$T_{max}=50$	1.4637	4.1835	2.0454
$T_{max}=75$	1.9404	6.9839	2.6427
$T_{max}=100$	2.2223	7.7108	2.7768
$T_{max}=125$	2.8059	14.8741	3.8367
$T_{max}=150$	2.8089	14.7204	3.8567

**Table 17:** Ablation study on maximum iterations.

Maximum Iterations	MAE (pop=30)	MSE (pop=30)	RMSE (pop=30)
$G_{max}=40$	0.49700	0.35266	0.59385
$G_{max}=45$	0.38241	0.22795	0.47744
$G_{max}=50$	0.36848	0.19665	0.44345
$G_{max}=55$	0.61054	0.57563	0.75870
$G_{max}=60$	1.18730	1.12210	1.76690

**Table 18:** Ablation study on population size.

Population Size	$\mathrm{MAE}\;(G_{max}=50$)	$\mathrm{MSE}\;(G_{max}=50)$	$\mathrm{RMSE}\;(G_{max}=50$)
pop=20	0.65755	0.56795	0.75362
pop=25	0.56288	0.51822	0.71988
pop=30	0.36848	0.19665	0.43345
pop=35	0.39902	0.24134	0.49126
pop=40	0.43038	0.28295	0.53193

## Data Availability

Data can be provided upon request.
